# From cyst to resection: a novel robotic approach to calcified hepatic hydatid disease

**DOI:** 10.1097/RC9.0000000000001095

**Published:** 2026-10-14

**Authors:** Ghader Jamjoum, Hasan Nassr, Basmah Abdulhadi

**Affiliations:** a Department of Surgery, Faculty of Medicine, King Abdulaziz University, Jeddah, Saudi Arabia; b Department of General Surgery, King Abdulaziz University Hospital, Jeddah, Saudi Arabia; c General Surgery Department, Dr. Sulaiman Al Habib Hospital, Jeddah, Saudi Arabia; d Department of Medicine, King Abdulaziz University, Rabigh, Mecca Province, Saudi Arabia

**Keywords:** hepatobiliary, hydatid cyst, parasitic infection, robotic surgery

## Abstract

**Introduction and importance:**

Cystic echinococcosis is a parasitic disease that commonly affects the liver and remains highly prevalent in endemic regions. While simple hepatic hydatid cysts can be managed conservatively, complex cysts may require surgical intervention. Although open or laparoscopic approaches are usually the preferred options for resection, factors such as large size, calcification, or anatomically challenging lesions often limit the possibility of complete surgical resection. Moreover, robotic-assisted surgery has emerged as a potential alternative. We report, to our knowledge, the first case of robotic-assisted partial hepatectomy for a calcified hepatic hydatid cyst using the Hugo robotic-assisted system (RAS) with a novel trocar configuration.

**Case presentation:**

A 42-year-old woman presented with a history of worsening right upper quadrant abdominal pain for 2 years. Imaging revealed a large (8.5 cm) calcified hydatid cyst involving hepatic segments V and VIII, with no biliary or vascular communication. Following preoperative albendazole therapy, the patient underwent robot-assisted right partial hepatectomy with cholecystectomy using the Hugo RAS with a novel four-trocar configuration. The procedure was completed successfully without conversion. The postoperative course was uneventful, and the patient was discharged on postoperative day 3.

**Discussion:**

This case highlights the feasibility of robotic-assisted resection in complex hepatic hydatid disease. The robotic approach provided enhanced visualization and retraction, facilitating safe dissection in a challenging anatomical location. The decision to perform anatomical resection was due to the cyst’s calcified nature, size, and location, which limited the use of laparoscopic or open approaches.

**Conclusion:**

Robotic-assisted partial hepatectomy using the Hugo RAS appears to be a safe option for selected cases of complex hepatic hydatid cysts. This case contributes to and raises hopes for the expanding role of robotic surgery in benign hepatobiliary disease.

1.

## Introduction

Cystic echinococcosis (CE), also called hydatid disease, is a parasitic infection caused by *Echinococcus granulosus* larvae^[^1^].^ Its life cycle involves dogs as definitive hosts, where the tapeworms reside in the intestines. Hoofed animals such as sheep, goats, and cattle serve as intermediate hosts in which the larvae grow. Humans may accidentally become intermediate hosts by ingesting food, water, or soil contaminated with echinococcal eggs found in dog feces^[^2^]^. After ingestion, the eggs hatch, penetrate the intestinal wall, and reach the liver through the portal circulation, where they develop into hydatid cysts. About 70% of these cysts inhabit the liver^[^3^]^.

HIGHLIGHTS

- First reported use of the Hugo robotic-assisted system in a hepatic hydatid cyst.
- Robotic partial hepatectomy for a calcified complex lesion.
- Novel trocar configuration improves surgical access.
- Safe resection without conversion or complications.
- Expanding the robotic role in benign hepatobiliary surgery.

Previous studies have shown that human CE is highly prevalent in the Middle East and North Africa^[^4,5^]^. This can be attributed to the abundance of cattle and sheep, which are an integral part of the region's natural resources, given their economic importance. While human CE can be asymptomatic and can be managed with available medical therapy or percutaneous approaches, complex cysts, characterized by large size, multiseptated walls, inaccessible locations, or biliary communication, most commonly necessitate surgical intervention^[^6^]^.

Minimally invasive approaches to CE have advanced in recent years. Although laparoscopic hepatic resection is possible, factors such as cyst size, complexity, or inaccessible locations can limit instrument articulation. Therefore, robotic surgery is gaining favor for such cases. To our knowledge, following a structured search of PubMed, Scopus, and Google Scholar (search terms: “robotic,” “Hugo RAS,” “hydatid cyst,” “echinococcosis,” “hepatectomy”), several robotic-assisted resections for hepatic hydatid disease have been reported using the da Vinci robot; however, we identify no prior report of anatomical resection for a calcified hydatid cyst performed with the Hugo robotic-assisted system (RAS) using the unique new trocar positioning described in this case. This paper is reported in line with the latest SCARE criteria.

## Case report

A 42-year-old Saudi woman, known to have a liver cyst, presented to the general surgery clinic with a 2-year history of intermittent right upper quadrant abdominal pain, with increasing severity at the time of presentation. She reported episodes of subjective pallor and mild jaundice. Her past surgical history is significant for one cesarean section 3 years ago. She has no known drug allergies. Additionally, her social history indicated that she resides in an urban area with no recent animal contact. On initial physical examination, the patient was hemodynamically stable and appeared comfortable. Her abdominal examination revealed central adiposity and a soft, non-tender abdomen.

Initial laboratory tests showed elevated aspartate aminotransferase (396 U/L), alanine aminotransferase (181 U/L), and leukocytosis (white blood cell count of 18.2 × 10^9^*/*L), with all other tests within normal ranges. A computed tomography scan (CT) triphasic liver scan (Fig. 1) revealed diffuse decreased attenuation of the liver, in keeping with diffuse fatty infiltration, and a sizable, rounded cystic lesion with heterogeneous content and a heavily calcified wall located in hepatic segment VIII/V, measuring 7 × 6.5 × 8.5 cm in the anteroposterior, transverse, and craniocaudal dimensions, respectively. It contains multiple curvilinear hyper-attenuating internal structures in favor of a hydatid cyst. Consequently, *Echinococcus granulosus* serology was ordered and returned positive. Subsequently, an abdominal magnetic resonance imaging (Fig. 2) was performed for more comprehensive assessment and possible surgical intervention. This confirmed a 7.9 × 6.1 × 7 cm round, well-defined liver lesion occupying segments 5, 6, 7, and 8, with internal heterogeneous components and a low T2 and T1 rim in keeping with calcification on the CT scan, which is suggestive of a calcified hydatid cyst. There is no evidence of communication between the liver lesion and the biliary system or the hepatic vessels. No evidence of biliary dilatation. The hepatic vessels are patent. There is mild diffuse fatty infiltration of the liver, with a small, round, focal fat-sparing area in segment seven medially, which demonstrates enhancement in the arterial phase. No other focal liver lesions. A dependent filling defect is seen within the gallbladder and could represent a tiny stone or sludge.

**Figure 1.**
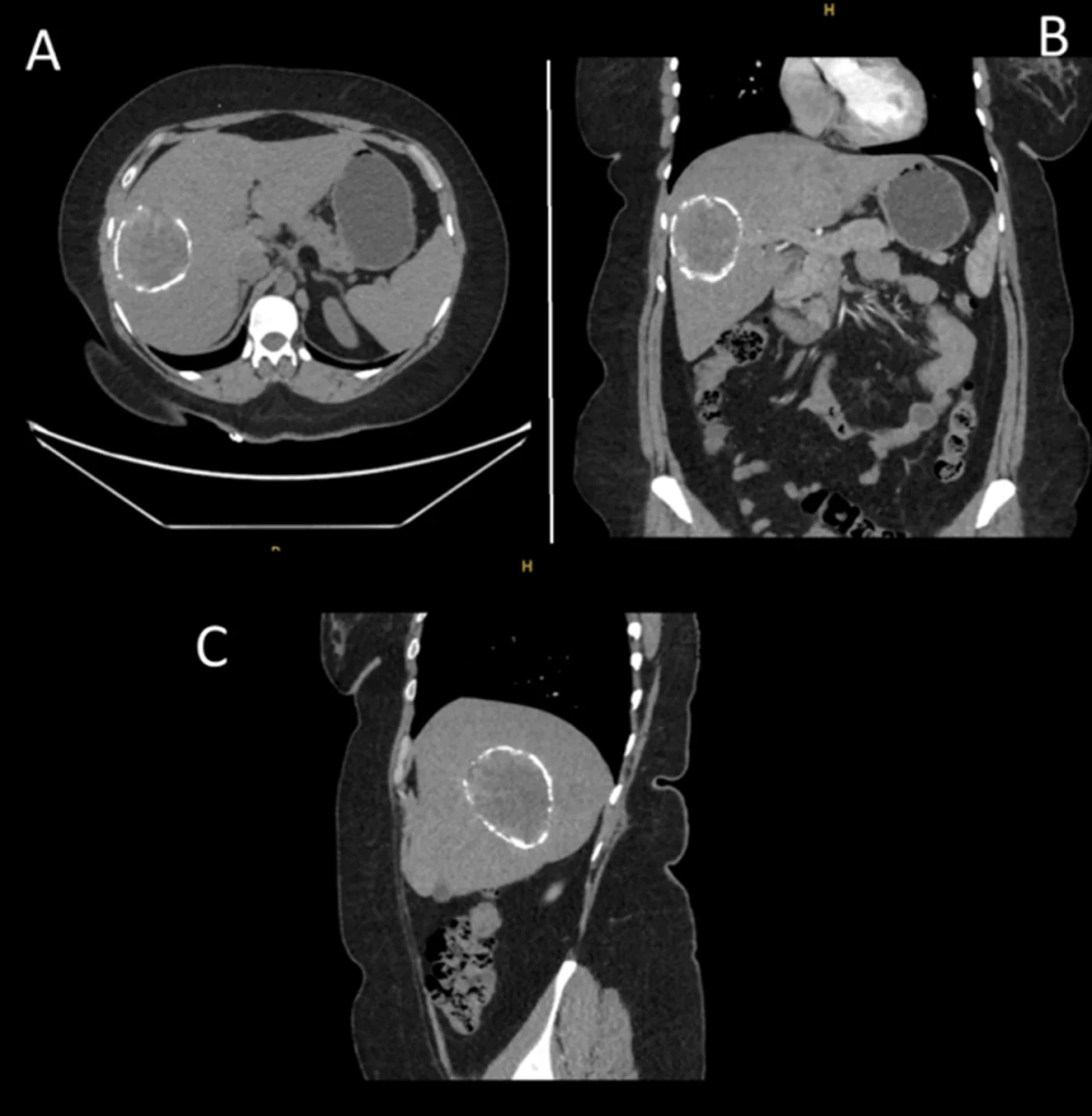
Triphasic CT liver scan. Axial (A), coronal (B), and sagittal (C) sections demonstrate a heavily calcified hydatid cyst wall in segments V and VIII, with no evidence of biliary or vascular involvement.

**Figure 2.**
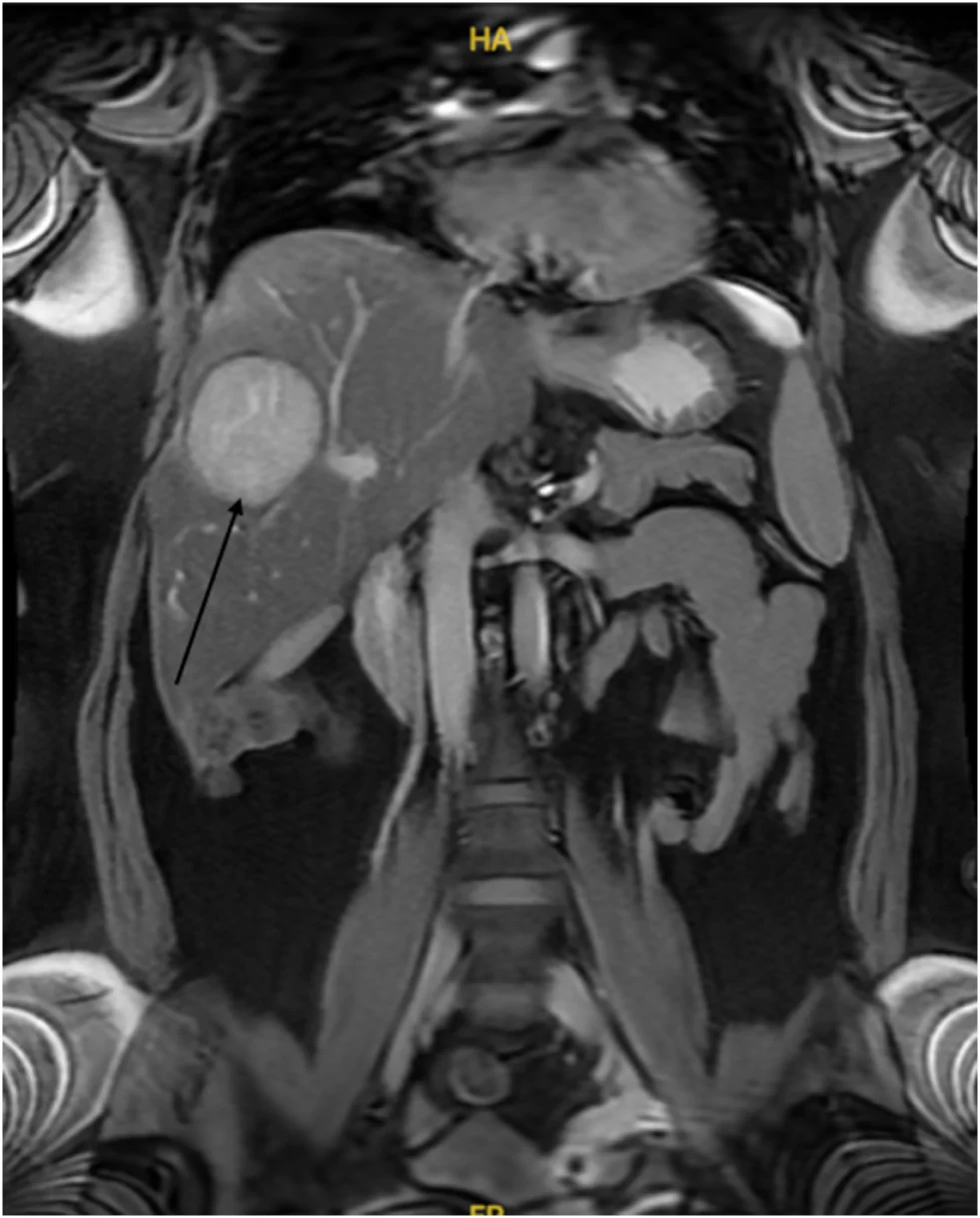
Abdominal MRI. The arrow indicates the rim of a 7.9 × 6.1 × 7 cm liver lesion, corresponding to the calcified wall seen on CT.

After multidisciplinary discussion and obtaining the patient’s consent, the plan was a 4-week course of albendazole therapy (400 mg twice daily), followed by a robotic right partial hepatectomy. Albendazole was administered preoperatively as a precautionary measure to reduce the theoretical risk of viable parasitic dissemination in the event of intraoperative spillage. All risks and possible complications of the operation were explained thoroughly to the patient. The operation was performed under general anesthesia along with epidural analgesia. The patient was placed in the supine position with legs split. The bed was tilted 15 degrees upward, then rolled 15 degrees upward from the left shoulder. Trocars were then placed manually, and pneumoperitoneum was established. The unique four-trocar configuration (Fig. 3) was as follows:

**Figure 3.**
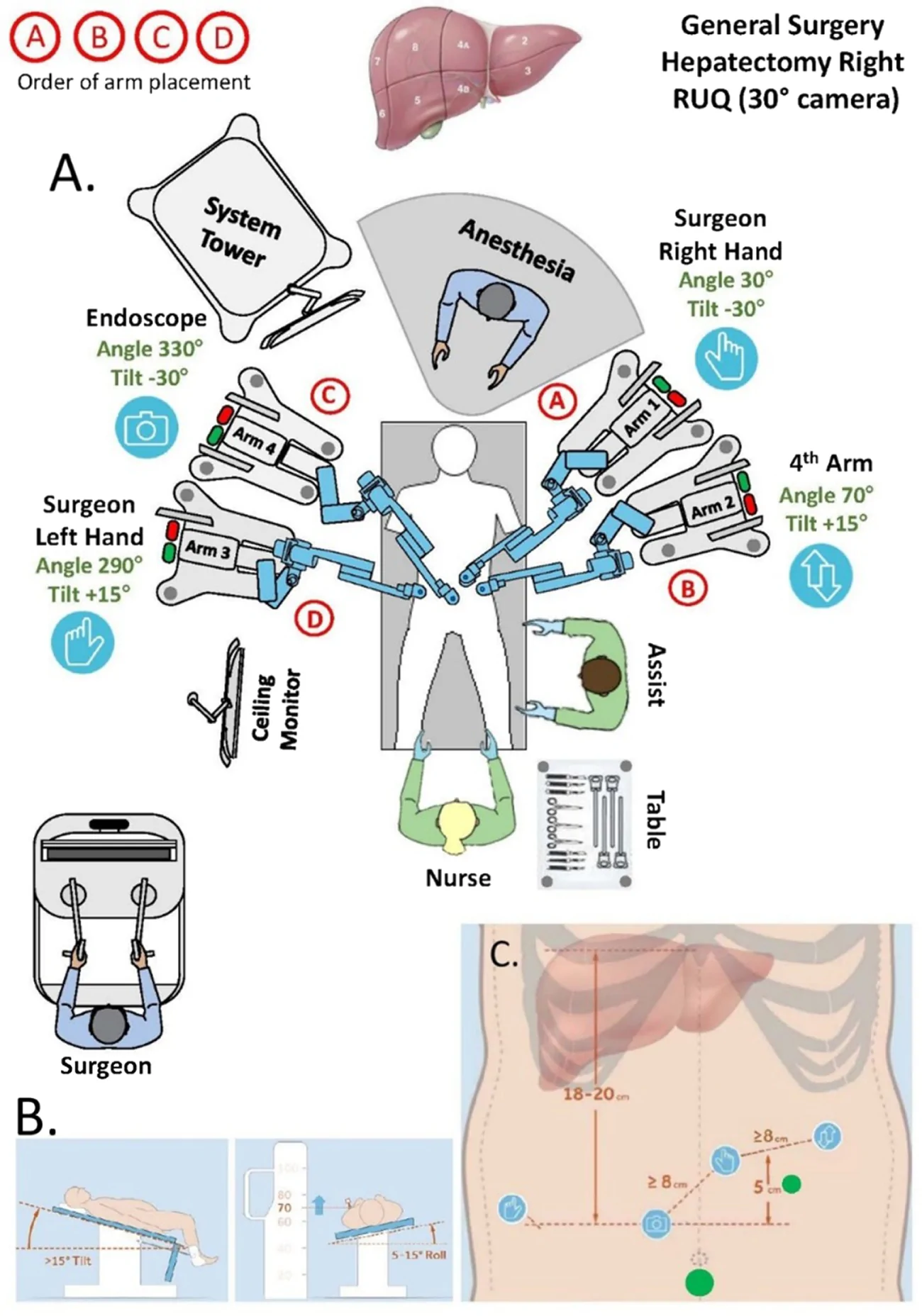
(A) The depicted operating room setup for each team member, along with the docking of the Hugo arms, the monitor, and the tower. (B) The patient’s positioning on the table. (C) The unique 4-trocar configuration.

A 12 mm optical port was placed supraumbilically.

- Three 8 mm robotic trocars were positioned (below the left subcostal arch at the midclavicular line, the epigastric region between the optical port and the left subcostal port, and at the right hypochondrium) for the surgeon’s left, right, and fourth robotic arms.

The Hugo RAS system was then docked at the patient’s right shoulder (Supplemental Digital Content Figure S1, available at: https://links.lww.com/IJSCR/A188). Upon initial exploration, the cyst was visualized. The liver was fully mobilized. Under direct vision, four sponges soaked in hypertonic saline were inserted and used to open the cystic structure. All intracystic fluid and daughter cysts were successfully aspirated. The cyst wall was then removed using a combination of monopolar scissors and bipolar cautery. Anatomical resection of segments V and VIII was then performed. Finally, a standard cholecystectomy was performed, given the preoperative findings on imaging. Excellent hemostasis was achieved with proper irrigation and application of Arista and Hemopatch. The surgery was completed robotically without the need for an open conversion. The operative time was approximately 3 hours. Estimated blood loss was approximately 700 mL. No vascular inflow occlusion was required during liver transection. There were no intraoperative complications.

The patient was discharged on the third postoperative day with an uneventful postoperative course. At the 1- and 2-week postoperative follow-up visits, the patient was doing well, with well-healed incisions. Final histopathological examination confirmed a calcified hepatic hydatid cyst with no evidence of malignancy.

## Discussion

The case discussed above, to our knowledge, is the first documented use of the Hugo RAS for the anatomical surgical resection of a hepatic hydatid cyst. The calcified nature of the cyst, its large size (8.5 cm), and the location at segments VIII/V posed a surgical challenge during the procedure. On the other hand, several advantages were provided, including the extensive articulation of the surgical instruments, optimal 3D magnification, and steady retraction using the third and fourth arms, ensuring the safe dissection of the calcified cyst from the hepatic wall. Hence, trocar configuration and proper planning are essential aspects in the context of robotic surgery. Evidently, the newly applied trocar positioning was a critical part of the surgery’s success, given the accessibility that this unique distribution provided, ensuring excellent triangulation of each arm, allowing access to the dome of the liver. Moreover, this minimized arm clashes and enhanced the use of the surgery’s established ergonomics.

The use of robotic surgery is increasing in the hepatobiliary setting, especially in the management of both malignant and certain benign lesions. However, its application in parasitic liver diseases remains limited due to the widespread use of open or laparoscopic approaches. This can be explained by concerns about intraoperative cyst rupture, spillage, or technical difficulties in resecting selected lesions. Our technique differs from a simple hydatid cyst evacuation; we successfully performed a right partial hepatectomy with a concomitant cholecystectomy. This highlights the evolving role of the use of robot-assisted devices in performing complex, parenchymal-sparing liver resections^[^7^]^. Open surgery remains the standard approach for many complex hepatic hydatid cysts because it provides wide exposure and facilitates management of large lesions or biliary communications. However, it is associated with greater postoperative pain, a longer hospital stay, and delayed recovery. Conventional laparoscopy offers the benefits of minimally invasive surgery but may be technically challenging in large, calcified, or posterosuperior hepatic lesions because of limited instrument articulation^[^8^]^. Robotic liver surgery has also been successfully used for a wide spectrum of benign and malignant hepatic lesions, with outcomes comparable to conventional minimally invasive approaches in appropriately selected patients. Nevertheless, careful patient selection remains essential, and conventional laparoscopic or open surgery continues to be the preferred option in many straightforward cases.

In the context of our case, although calcified hydatid cysts are often considered inactive, surgical intervention was considered appropriate because the patient remained symptomatic with progressively worsening right upper quadrant pain. Furthermore, the lesion measured approximately 8.5 cm, demonstrated extensive calcification, and involved segments V and VIII, making conservative procedures less favorable^[^8^].^ Fortunately, our case highlights the potential of robotics to expand the surgical options for complex hepatic hydatid disease, particularly in carefully selected patients requiring delicate and controlled resection.

## Conclusion

The limitations of RAS use stem from the lack of numerous comparative studies to closely address long-term outcomes, cost-benefit analyses, and possible outcomes compared with laparoscopic interventions. Moreover, it is essential to compare different types of RAS systems to determine whether outcomes would truly vary. In conclusion, RAS liver resection using the Hugo is a feasible option for surgically excising complex hydatid cysts, and this case adds to the initial evidence supporting the expansion of such a novel robotic platform into complex hepatobiliary surgeries for benign disease.

## Data Availability

Not applicable.

## Research registration unique identifying number (UIN)

This case report does not require registration as a research study.

## References

[1] Eckert J Deplazes P. Biological, epidemiological, and clinical aspects of echinococcosis, a zoonosis of increasing concern. Clin Microbiol Rev 2004;17:107–35. 10.1128/CMR.17.1.107-135.2004 PMC321468 14726458

[2] Agudelo Higuita NI Brunetti E McCloskey C. Cystic echinococcosis. J Clin Microbiol 2016;54:518–23. 10.1128/JCM.02420-15 PMC4767951 26677245

[3] Botezatu C Mastalier B Patrascu T. Hepatic hydatid cyst - diagnose and treatment algorithm. J Med Life 2018;11:203–09. 10.25122/jml-2018-0045 PMC6197524 30364592

[4] Sadjjadi SM. Present situation of echinococcosis in the Middle East and Arabic North Africa. Parasitol Int 2006;55 Suppl:S197–S202. 10.1016/j.parint.2005.11.030 16337429

[5] Abdulhameed MF Habib I Al-Azizz SA. A retrospective study of human cystic echinococcosis in Basrah province, Iraq. Acta Trop 2018;178:130–33. 10.1016/j.actatropica.2017.11.011 29183851

[6] Pakala T Molina M Wu GY. Hepatic echinococcal cysts: a review. J Clin Transl Hepatol 2016;4:39–46. 10.14218/JCTH.2015.00036 PMC4807142 27047771

[7] Nota CL Rinkes IHB Molenaar IQ. Robot-assisted laparoscopic liver resection: a systematic review and pooled analysis of minor and major hepatectomies. HPB 2016;18:113–20. 10.1016/j.hpb.2015.09.003 PMC4814602 26902129

[8] Brunetti E Kern P Vuitton DA. Expert consensus for the diagnosis and treatment of cystic and alveolar echinococcosis in humans. Acta Trop 2010;114:1–16. 10.1016/j.actatropica.2009.11.001 19931502

